# Interest in complementary and alternative medicine among participants in a study on cancer prevention by green tea extract – results from an expert-based survey of MIRACLE trial participants

**DOI:** 10.1186/s12906-025-05087-3

**Published:** 2025-10-02

**Authors:** Anna Melzer, Niklas Sturm, Friederike Rohlmann, Rainer Muche, Julia Stingl, Thomas J. Ettrich, Thomas Seufferlein

**Affiliations:** 1 Department of Internal Medicine I, Ulm University Hospital, Ulm, Germany; 2 Institute of Epidemiology and Medical Biometry, Ulm University, Ulm, Germany; 3 Department of Clinical Pharmacology and Pharmacoepidemiology, Heidelberg University Hospital, Heidelberg, Germany

**Keywords:** Conventional medication, Complementary and alternative medicine, Survey, Factor analysis, Cancer prevention, Green-tea extract

## Abstract

**Objective:**

Although chemopreventive strategies such as acetylsalicylic acid have shown potential in cancer prevention, they also entail adverse effects. This leads to growing interest in “natural” compounds, such as plant-based extracts, which may offer preventive benefits with fewer side effects. However, little is known aboutindividuals' personal attitudes towards conventional medications and complementary or alternative therapies when they are eligible for various cancer screening and prevention programmes. The present study aims to investigate the attitude toward medications and various therapies, as well as their prescription, among subjects participating in a clinical trial on the prevention of colorectal adenomas using green tea extract. A further goal was to assess whether the study predominantly attracted individuals with a strong preference for natural products or a more diverse set of opinions.

**Methods:**

992 participants (age 50–80 years) of the MIRACLE study (a randomised study comparing green tea extract vs. placebo for secondary prevention of colorectal adenomas, NCT01360320) in 40 study centres across Germany received a 25-item, expert-based questionnaire regarding their attitude towards different medications and therapies including complementary and alternative medicine. Basic characteristics including age, gender, ECOG status, and lifestyle factors were also recorded. An exploratory factor analysis was used to group the items into eight thematic areas, which were then analysed for potential associations with the participants’ baseline characteristics.

**Results:**

Response rate to the questionnaire was 94.3% (935/992). Survey participants mainly expressed positive attitudes towards both conventional and complementary medicine. Most were open to genetic testing for personalised treatment. Comparing the categories of the exploratory factor analysis revealed that female gender and regular exercise were associated with the regular use of alternative healing methods (i.e. acupuncture or homeopathic medicine). A more favourable view of alternative approaches was more common among younger and better-educated individuals, but the same groups also rated conventional medicine less negatively.

**Conclusion:**

The findings of this study reveal a relatively high level of openness towards both conventional and alternative medicine among participants of the MIRACLE trial, as well as certain features, such as age, gender, and education that impact on the attitude towards medications and therapies among participants in a study on secondary prevention of colorectal polyps by green tea extract.

**Trial registration:**

ClinicalTrials.gov: NCT01360320.

**Supplementary Information:**

The online version contains supplementary material available at 10.1186/s12906-025-05087-3.

## Background

Complementary and alternative medicines (CAM) include healing methods and resources from different health systems, practices and cultural backgrounds [1]. There is a relatively high and, in some cases, increasing interest in CAM both in Germany [2, 3] and globally [4–7]. Socio-demographic studies show that the average CAM user tends to be affluent, educated, Caucasian, female and middle-aged. A particularly high interest in CAM is observed in cancer patients [8, 9]. A meta-analysis of 152 studies with over 65,000 cancer patients showed a prevalence of current use of CAM of around 40% [10]. Reasons for this attitude could include a desire to improve quality of life, reduce the side effects of oncological therapy or seek spiritual healing [11]. There is insufficient data on the attitudes or interests of healthy individuals towards using CAM or conventional drugs in the context of cancer prevention. Indeed, various compounds in the CAM spectrum fulfil requirements of chemopreventive agents: good tolerability, few side effects, simple application, uncomplicated and inexpensive access [12] and a potentially preventive effect demonstratable by means of evidence-based medicine [13]. Amongst conventional drugs ASA is the most extensively studied drug for colorectal cancer prevention to date [14].

The aim of the present study was to explore the attitude towards medication, various therapies (including CAM) and their prescription in a specific group of people, participants of the MIRACLE trial, that studied the use of green tea extract for the secondary prevention of colorectal adenomas (NCT01360320) [13]. We were interested in the general attitude of trial participants towards different types of treatment, given their obvious interest in cancer prevention, and whether the trial topic would particularly appeal to people prone to complementary and alternative medicine (CAM).

## Methods

The MIRACLE trial (Minimising the Risk of Metachronous Adenomas of the Colorectum with Green Tea Extract, NCT01360320) was an interventional, prospective, randomised, placebo-controlled, double-blind study funded by the German Cancer Aid (grant number 109122). It investigated the effects of 150 mg of green tea extract twice daily (total daily dose: 300 mg), compared to placebo on the incidence of metachronous colorectal adenomas over a period of three years in a Caucasian population for colorectal cancer screening [13]. Flyers and posters were used to promote participation in the MIRACLE study. Participants were informed that the capsules containing green tea extract were classified as a dietary supplement according to the food legislation, but not as a pharmaceutical according to the pharmaceutical law. At the time of inclusion in the study, the participants received an additional questionnaire with the primary objective of getting to know their attitude towards medications and therapies, including conventional medicine and CAM as well as their attitude towards the prescription of medication. Filling in the questionnaire was not a direct part of the MIRACLE trial and therefore not compulsory for participation in the trial. The secondary objectives of the project included the investigation of possible associations of attitudes with baseline characteristics such as age, gender, place of residence, level of education and physical activity. Finally, we were interested whether participation in a trial that used green tea extract for three years for secondary prevention of colorectal adenomas would select a population particularly prone to CAM.

All 992 subjects at 40 German study centres (clinics and practices) who gave their consent to participate in the MIRACLE study were asked to complete a questionnaire for inclusion in the study. Inclusion and exclusion criteria in MIRACLE were reported previously [13] and these were identical for participation in the associated survey. In brief, all participants were between 50 and 80 years of age, had a history of a colorectal adenoma removed within 6 months prior to study recruitment and should receive green tea extract or placebo over a period of three years, after which a follow up colonoscopy was scheduled. The questionnaire contained 25 questions on personal attitudes toward medications and therapies and their prescription (Additional file 1). The answers corresponded to a 5-point Likert scale from 1 = strongly disagree to 5 = strongly agree. All participant data were pseudonymised.

The baseline characteristics collected and published as part of the MIRACLE trial [13] are listed in Additional file 2.

Based on the baseline characteristics (see Additional file 2) and additional parameters collected in the context of the MIRACLE trial (including origin from Germany [yes/no], place of residence [East, West, or South Germany], ECOG performance status [0, 1, or 2], and adherence during the study phase—defined as participation in the randomised phase for at least 28 months and intake of at least 1,401 capsules of green tea extract or placebo, respectively), a comparison was conducted to evaluate whether the survey participants (i.e. all MIRACLE trial participants who returned the questionnaire with at least one answered item) differed relevantly from the overall MIRACLE study population. In a next step, several baseline characteristics were further examined whether they were associated with particular attitudes in the survey. These included: gender, age, intake of low-dose ASA, number of removed colorectal adenomas > 1 cm, place of residence, regular exercise, highest educational qualification, and adherence. Two of the characteristics were further subdivided: age (< 55 years vs. ≥ 55–74 years vs. ≥ 75 years) and regular exercise (no regular exercise vs. regular exercise ≤ 3 h per week vs. regular exercise > 3 h per week).

### Statistical analysis

All data were analysed descriptively. Absolute and relative frequencies are given for categorical variables, while the mean and standard deviation are shown for continuous variables. Questionnaires were described as “completed” if at least 1 question was answered. “Not completed” meant that 0 questions were answered which was synonymous with non-participation in the survey.

An exploratory factor analysis with varimax rotation was conducted to identify common features among the 25 ordinal-scaled items in the questionnaire. The number of factors was determined using the eigenvalue rule [15]. Based on this criterion, six factors with eigenvalues above 1 were initially extracted. To control for a possible selection bias, multiple imputation (Fully Conditional Specification (FCS) discrim method [16], 5 repetitions) was performed, after which the factor analyses were repeated. This resulted in eight factors, for which Cronbach’s alpha was calculated to assess internal consistency. Table 1 displays the combinations of questions that produced the highest Cronbach’s alpha values, summarised as these eight factors. Each factor underwent a plausibility check, and an appropriate content-related label was assigned.

**Table 1:** Results of the exploratory analysis of factors with corresponding Cronbach’s alpha

Factor	Question numbers	Label	Cronbach’s alpha
1	13, 14, 15, 16	Increased/negative reaction to conventional medication	0.79
2	21, 23, 25	Positive attitude towards alternative healing methods	0.71
3	5, 18, 19, 20	Preference for herbal versus conventional medication	0.75
4	3, 6, 7, 10	Positive attitude towards conventional medication	0.54
5	1, 11, 12	Critical judgments about doctors’ medication practices	0.61
6	2, 4, 8, 9	Critical/negative evaluation of conventional medications	0.64
7	22, 24	Regular use of alternative treatments/dietary supplements	0.52
8	17	Genetic tests for personalized medication	/*

* Since Factor 8 only contained one question, no Cronbach's alpha can be generated in this case

For each factor, the mean value of the responses to the corresponding questions (based on a Likert scale from 0 = strongly disagree to 5 = strongly agree) was calculated and used for further analysis. If only a small number of responses were missing (maximum of one missing response for factors with three items, or two for factors with four items), the mean was calculated based on the available responses. If more than this number of responses were missing, the entire factor was classified as missing. The results of the analysis of the eight factors are presented for each category of the baseline characteristics, including minimum, median, quartiles, maximum, as well as the mean and standard deviation based on the Likert scale values. For comparisons between two categories (e.g. gender: female vs. male), an unpaired t-test was used. Where more than two categories were compared, analysis of variance (ANOVA) followed by pairwise comparisons was applied. The significance level for all tests was set at alpha = 5%. As this was a purely exploratory evaluation, no adjustment was made for multiple testing [17]. All analyses were performed with the statistical software SAS 9.4 (SAS Institute Inc., Cary, NC) and GraphPad Prism Version 10.0.0 for Windows (GraphPad Software, Boston, Massachusetts USA). R (R Core Team, 2024) was used to visualise the distribution of the different answer options according to the Likert scale.

## Results

### Participation rate in the survey

Of the 992 participants in the MIRACLE study who provided written informed consent, 935 (94.3%) completed the questionnaire at least partially (referred to as “completed”), 5.7% did not complete the questionnaire. Among these 935 completed questionnaires, there were 125 (13.4%) with 1–2 unanswered questions, 17 (1.8%) with 3–10 unanswered questions and 3 (0.3%) with > 10 unanswered questions. Baseline characteristics and questionnaire completion status are presented in Table 2.

**Table 2:** Baseline characteristics and questionnaire completion status

Baseline characteristic	Category	Total, *n*	Questionnaire completed, *n* (%)	Questionnaire not completed, *n* (%)
Gender	Male	616	578 (93.8)	38 (6.2)
	Female	376	357 (94.9)	19 (5.1)
Age	< 65 y	493	471 (95.5)	22 (4.5)
	≥ 65 y	499	464 (93.0)	35 (7.0)
Low-dose ASA	Yes	162	148 (91.4)	14 (8.6)
	No	830	787 (94.8)	43 (5.2)
Adenoma > 1 cm	Yes	281	265 (94.3)	16 (5.7)
	No	616	588 (95.5)	28 (4.5)
	n. a.	95	82 (86.3)	13 (13.7)
Origin from Germany	Yes	944	906 (96.0)	38 (4.0)
	No	25	23 (92.0)	2 (8.0)
	n. a.	23	6 (26.1)	17 (73.9)
Place of residence	East Germany*	226	211 (93.4)	15 (6.6)
	West Germany**	356	330 (92.7)	26 (7.3)
	Southern Germany***	410	394 (96.1)	16 (3.9)
ECOG	0	962	909 (94.5)	53 (5.5)
	1	25	23 (92.0)	2 (8.0)
	2	3	2 (66.7)	1 (33.3)
	n. a.	2	1 (50.0)	1 (50.0)
Regular exercise	Yes	743	715 (96.2)	28 (3.8)
	No	239	216 (90.4)	23 (9.6)
	n.a.	10	4 (40.0)	6 (60.0)
Educational qualification	None****	3	2 (66.7)	1 (33.3)
	Lower sec. school	351	351 (100.0)	0 (0.0)
	Intermed. sec. school	273	272 (99.6)	1 (0.4)
	High school	61	61 (100.0)	0 (0.0)
	University degree	241	239 (99.2)	2 (0.8)
Alcohol consumption	Yes	415	391 (94.2)	24 (5.8)
	No	569	542 (95.3)	27 (4.7)
	n. a.	8	2 (25.0)	6 (75.0)
Nicotine consumption	Never	426	401 (94.1)	25 (5.9)
	Current	169	163 (96.4)	6 (3.6)
	Former	380	360 (94.7)	20 (5.3)
	n. a.	17	11 (64.3)	6 (35.3)
Adherence	Yes	912	864 (94.7)	48 (5.3)
	No	80	71 (88.8)	9 (11.2)

The percentages in brackets refer to the total number of participants in the respective categories

*Abbreviations:* *ASA* Acetylsalicylic acid, *ECOG* Eastern Cooperative Oncology Group performance status, *Intermed* Intermediate, *N* Number, *n. a * Not available, *SD* Standard deviation, *Sec*. Secondary, *Y* Years

* Mecklenburg-Western Pomerania, Saxony-Anhalt and Berlin, **North Rhine-Westphalia, Schleswig-Holstein, Lower Saxony and Hesse, *** Baden-Wuerttemberg and Bavaria, **** no school leaving certificate

The vast majority of respondents (790/935) answered every question in the survey. Some differences were observed: individuals who reported engaging in regular physical activity were more likely to complete the questionnaire than those who did not. In addition, higher adherence during the whole MIRACLE trial was also associated with a higher rate of questionnaire completion than non-adherence (see Table 2).

### Distribution of responses to the 25 questions

**Fig. 1:**
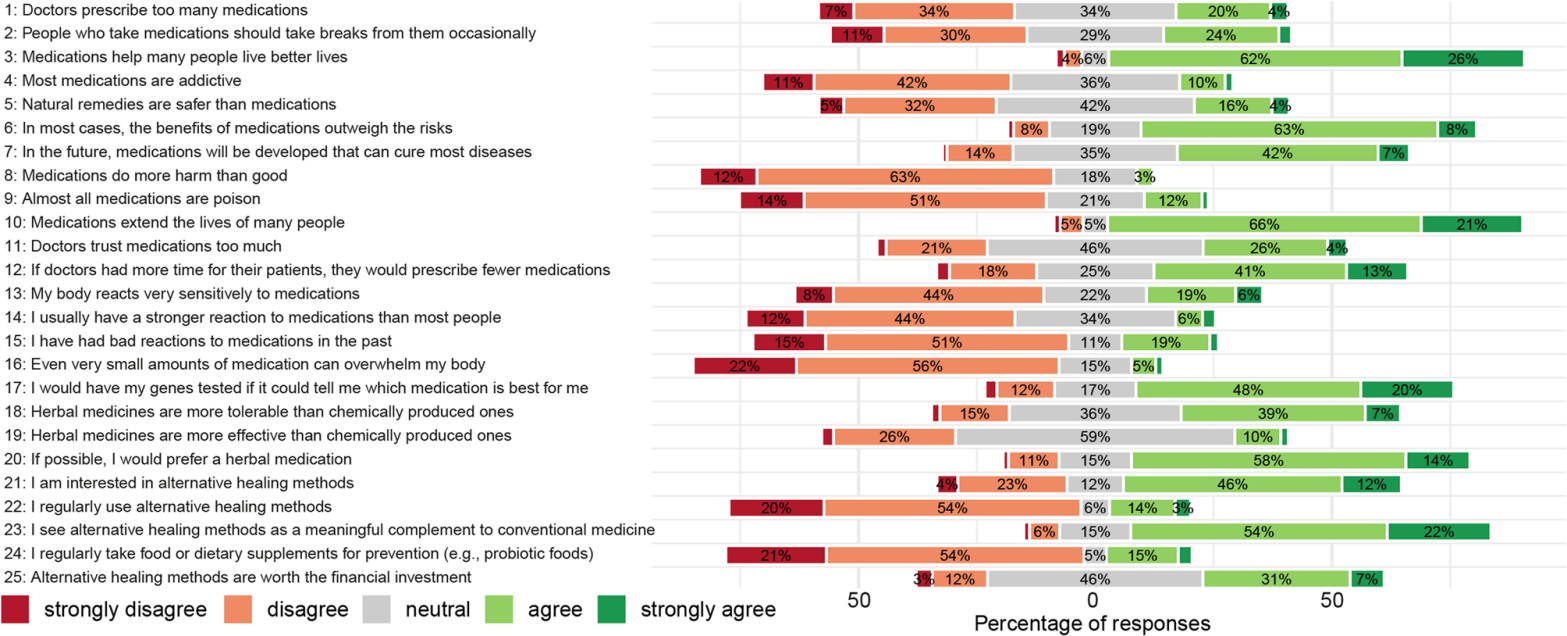
Distribution of the different answer options to the 25 questions on the 5-point Likert scale. The left-hand side of the image shows a slightly abridged version of the 25 questions asked, while the right-hand side shows the distribution of the answers to these questions across the 5 points of the Likert scale in the form of a diverging stacked bar chart. The scale ranged from strongly disagree (dark red) to disagree (light red), neutral (grey), agree (light green) and strongly agree (dark green). Percentages for each response category are displayed within the bars. In cases where the number of responses was very low and the bar correspondingly short, percentage labels were omitted for improved visual readability

Responses to the questionnaire reflected a predominantly positive attitude toward both conventional and CAM approaches and particularly toward herbal medicines (percentage distribution of responses see Fig. 1; additional absolute frequencies of responses see Additional file 3). Furthermore, a total of 87.8% of participants agreed with the statement that conventional medications can improve quality of life (question 10), and 87.7% agreed that they can help extend lifespan (question 3). Additionally, 71.0% rated the overall benefit–risk ratio of conventional medications as favourable (question 6), and 49.0% agreed that medications will be developed in the future that will cure most diseases (question 7). Only a minority (4.0%) were in agreement with the statement that conventional medicines do more harm than good (question 8), 13.0% agreed that almost all medicines are poison (question 9), and 11.2% that most medications are addictive (question 4). 20.4% of participants reported negative experiences with conventional medications in the past (question 15), and 8.4% indicated that they usually react more strongly to medication than most other people (question 14). Only 20% agreed that natural remedies are safer than medications, while 41.9% were neutral and 37.4% disagreed (question 5). A total of 74.0% of participants stated that they would consider undergoing genetic testing to help identify the most effective and tolerable medications based on their individual profile (question 17).

With regard to prescribing behaviour, 41.4% disagreed that doctors prescribe too much medication (question 1), whereas 54.6% agreed that doctors would prescribe less medication if they would spend more time with their patients (question 12). 30.4% agreed with the notion that doctors trust medications too much (question 11). Regarding herbal medicines, 71.5% agreed that they would prefer herbal medicines, if possible (question 20), and 46.3% agreed that herbal medicines are more tolerable than chemically produced ones (question 18). Only 11.3% agreed that herbal remedies are generally more effective than conventional medicines (question 19). 76.1% agreed that alternative healing methods are a meaningful complement to conventional treatment (question 23) and 58.7% agreed that they are interested in alternative healing methods (question 21). However, 74% of the participants disagreed when asked whether they used alternative healing methods regularly (question 22).

**Fig. 2:**
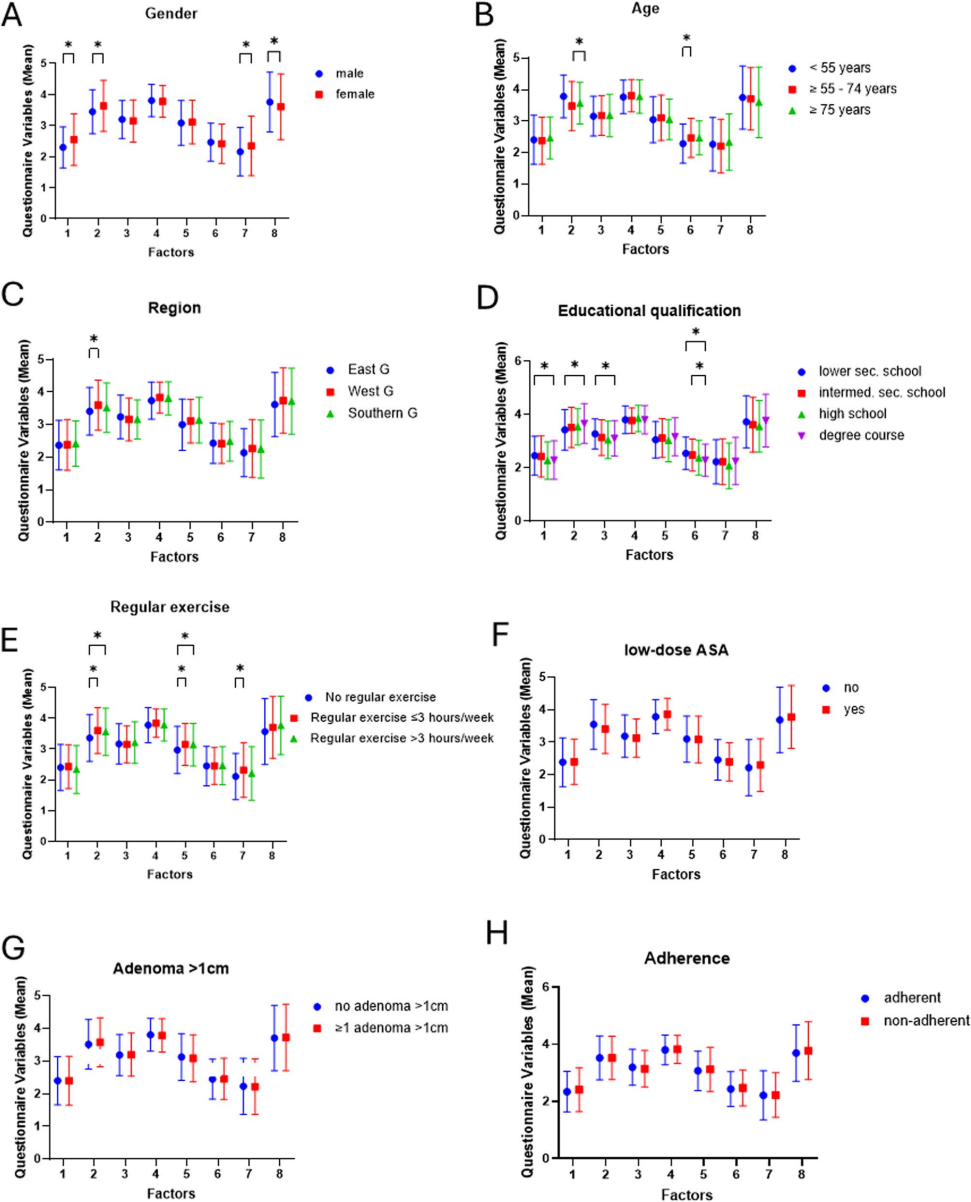
Results of the analysis of factors based on the participant characteristics examined. Legend: The participant characteristics examined include gender (**A**), age (**B**), region of the study centre (**C**), educational qualification (**D**), regular exercise (**E**), intake of low-dose ASA (**F**), presence of adenomas>1 cm in the initial colonoscopy (**G**), and adherence to study medication **H**. The eight factors—each representing a surveyed topic—are shown along the x-axis. Mean response values ± standard deviations are depicted on the y-axis using a 5-point Likert scale ranging from 1 (strongly disagree) to 5 (strongly agree). For improved clarity, the y-axis starts at 0, although values below 1 were not possible. Statistically significant differences between groups, identified via unpaired t-tests, are marked with an asterisk (*). *Abbreviations:* *ASA* Acetylsalicylic acid, *Intermed*. Intermediate, *Sec.* Secondary

Next, we performed an exploratory factor analysis to identify common features between the 25 ordinal scaled questionnaire items and their potential associations with baseline characteristics (Fig. 2**)**. For gender, differences in response behaviour between men and women were reflected in significantly different values on the Likert scale for Factors 1 (*p* < 0.001), 2 (*p* < 0.001), 7 (*p* = 0.002), and 8 (*p* = 0.040) (Fig. 2A): women tended to have a more negative attitude towards conventional medication (Factor 1 and a more positive view of alternative healing methods (Factor 2). They also reported using such methods and supplements more frequently (Factor 7). In contrast, men were more open to genetic testing to personalise treatment (Factor 8). Associations with specific factors were also evident in differences between age groups. (Fig. 2B): participants under 55 years of age displayed higher values for Factor 2 (*p* < 0.001), indicating a more favourable view of alternative medicine, and lower values for Factor 6 (*p* = 0.016), suggesting they were less critical of conventional treatments than those aged 55–74.In terms of study region, participants from West Germany showed higher values for Factor 2 (*p* = 0.015), indicating a more positive attitude towards alternative treatments compared to those from East Germany (Fig. 2C).

The highest educational qualification was significantly associated with Factors 1 (*p* = 0.038), 2 (*p* = 0.004), 3 (*p* = 0.001), and 6 (*p* < 0.001) (Fig. 2D): university graduates showed the strongest support for alternative remedies (Factor 2). Participants with lower secondary education more often expressed scepticism towards conventional medication (Factors 1 and 6) and preferred herbal over conventional medicines (Factor 3).

Regarding regular exercise, differences in exercise habits were reflected in the expression of Factors 2 (*p* = 0.001), 5 (*p* = 0.007), and 7 (*p* = 0.011) (Fig. 2E): individuals who exercised regularly had a more positive view of alternative treatments (Factor 2), used them and dietary supplements more frequently (Factor 7), and were more critical of doctors’ prescribing behaviour (Factor 5) than those who did not exercise. The strongest contrast was seen between not active participants and those who exercised up to 3 h per week. No significant differences in any of the eight factors were found regarding low-dose ASA, adenomas > 1 cm, and adherence during the study phase (Fig. 2F-H).

## Discussion

The main purpose of the study was to explore the attitude of participants of the MIRACLE trial towards conventional as well as CAM methods. The questionnaire was well accepted. 94.3% completed the questionnaire most likely because participants who at the same time were included in the MIRACLE trial were highly motivated and consequently, completion rate was independent from most of the baseline characteristics. Of note, a small but significant difference was observed in the completion of the questionnaires in the ‘regular exercise’ category. Participants who engaged in regular exercise demonstrated a higher propensity for completing the survey. In an US survey of 10,201 people using CAM, 34.9% stated that they were more motivated to exercise regularly thanks to CAM [18]. Whether, conversely, regular physical activity is associated with more frequent use of alternative healing methods should be investigated in further studies. Participants classified as ‘adherent’ during the study phase of the MIRACLE trial were also more likely to complete the questionnaire. However, this finding should be interpreted with caution, as the ’adherent’ group was more than ten times larger than the ’non-adherent’ group, leading to a skewed distribution of characteristics. The participants were a highly selected population who were all prepared to undergo a screening colonoscopy three years after randomisation. It can be assumed that the participants were generally open to conventional diagnostic methods. The prevalence of alcohol (41.8%) and nicotine consumption (17.0%) amongst participants was low in comparison with the overall German population. It is estimated that around 74.4% of Germans between 18 and 56 years of age reported drinking alcohol [19] and around 30% reported smoking in the year of the survey [20]. These figures could indicate that a particularly health-conscious part of the population participated in the trial. Furthermore, the possibility of false-low values resulting from the social desirability effect cannot be discounted. There are only subtle differences between respondents to the survey (with at least 1 question answered) and the total number of participants in the MIRACLE trial. The former may have been slightly fitter with exercise and less ASA intake. The overall results of the survey indicate that the survey participants tend to be open to both conventional and alternative medical approaches. Participants generally had a favourable perception of conventional medications, particularly regarding their benefits in improving quality of life and prolonging life expectancy. Most participants also expressed openness toward herbal medicines and CAM treatment options, reflecting a multifaceted and non-exclusive attitude toward different therapeutic approaches. The combination of conventional medicine and CAM is more prevalent in Western populations than in other countries [21], and this pattern appears to be reflected in the present study collective, even though the questions were not explicitly aimed at combining different therapeutic approaches. This may also explain why the participants were interested in the effect of green tea extract on the secondary prevention of colorectal adenomas and willing to take green tea extract or a placebo daily over a period of three years.

Given the participants’ considerable interest in genetic testing for individualised medication, it is likely that those surveyed will support innovative procedures for diagnostics and therapy. This finding is noteworthy, as personalised medicine is often considered more relevant to tech-savvy individuals. However, it could also reflect a particular selection of participants for the MIRACLE trial.

Only a minority of participants expressed critical views towards conventional medicine, including concerns about toxicity or overprescription, indicating a relatively high level of trust in the health care system. Of note, over half of the participants agreed that physicians might be inclined to prescribe less medication if they had more time for patients underscoring a nuanced perception of the prescribing process. A German study showed that time constraints during consultations can indeed influence prescribing behaviour—specifically regarding antibiotics—by encouraging physicians to prescribe more quickly to conclude appointments and avoid conflict [22].

We also wanted to analyse a potential association of response patterns with certain baseline characteristics of the participants. Our exploratory factor analysis revealed subtle differences in the response behaviour particularly with respect to gender, region, education, age and level of regular exercise. A possible explanation for gender-specific differences with a more positive attitudes to conventional medication (Factor 1), alternative healing methods (Factor 2) and use of CAM (Factor 7) of female participants lies in biological and psychosocial differences. Women are more frequently affected by adverse drug reactions [23] and drug interactions [24], which may turn them to alternative therapies. A more frequent use of alternative healing methods by women has described in other studies [8, 9]. Other possible explanations for this finding may be a higher level of health consciousness and a greater openness to CAM [25]. Both groups, but to a greater extent male than female participants, were prepared for genetic testing to optimise their medication (Factor 8) in agreement with similar findings from other studies [26]. Alternative healing methods (Factor 2) but also conventional medication (Factor 6) were more positively viewed by participants below 55 years of age compared to older participants demonstrating a great openness of particularly the “younger” participants for all types of treatment. Interestingly, participants from the two federal states in the east of Germany had a more reserved attitude towards CAM, possibly due to a lower supply of CAM and a lower number of so-called alternative practitioners in these parts of Germany [27] or due to a lower gross domestic product (GDP in these regions limiting the financial resources for CAM [28]. The level of education was associated with characteristics of some factors. Amongst the participants the proportion with secondary school or intermediate secondary school diploma as highest degree was slightly higher compared to German microcensus data of an age comparable cohort [29] in agreement with the finding that the participation rate in colorectal cancer screening in Germany is higher among individuals with a higher level of education [30].

Attitudes towards specific forms of CAM may differ depending on one’s level of education. Our survey revealed that participants with lower levels of education prefer herbal medicine, which may indicate a desire for ‘natural’ treatment options alongside established medicines, while remaining sceptical of alternative healing methods. Interestingly, there was no significant difference in the use of alternative healing methods or dietary supplements (factor 7) between the various educational groups in our survey. Furthermore, there was no preference for CAM among those with higher education, which is inconsistent with published studies [2, 9].

Regarding exercise, the most pronounced differences in the levels of Factors 2, 5, and 7 were found between non-regular and regular exercisers (up to 3 h per week). A more frequent use of alternative remedies by amateur and professional athletes has been observed previously [31]. CAM might be used in this particular group to prevent or treat injuries and increase performance, with the hope of achieving results with few or no side effects.

### Limitations

The present study utilised a non-validated questionnaire. There were no questions regarding more specific forms of CAM or previous experiences with CAM that might have enabled a more detailed characterisation of the study population. We did not ask specific questions about phytotherapy. Regarding exercise, the item was solely quantified by time and not by training intensity. For example, the metabolic equivalent task (MET) method [32] could have been utilised. However, the focus of the survey was not on a very detailed analysis or on psychometric measurements, but rather on capturing a broad and practical picture of personal attitudes towards medication, therapies and alternative remedies in a specific study population. A further limitation is the single time point of the survey. An additional survey at the end of the randomised MIRACLE study phase would have enabled the assessment of potential changes in attitudes over time. The exploratory factor analysis with Varimax rotation allowed for the reduction of the 25 items into a smaller number of meaningful factors, simplifying interpretation and revealing underlying patterns in the data. However, a Cronbach’s alpha < 0.7 for the investigated Factors 4–8 may be an indication that the chosen scale may not be sufficiently reliable, meaning that the items within those four factors may not correlate strongly enough with each other [33, 34]. Thus, we conclude that the results of the exploratory factor analysis are of limited significance. For this reason, they are listed as a subordinate result. Finally, the cross-sectional nature of the survey does not allow for causal conclusions to be drawn. The use of self-report measures can also lead to bias due to social desirability.

In conclusion, this survey on attitudes towards medications and therapies and their prescription among individuals aged 50 to 80 who participated in a clinical trial on secondary prevention of colorectal adenomas with green tea extract was characterised by a high willingness to participate. It revealed a relatively high level of openness amongst participants toward both conventional medicine and CAM. Looking into various factors associated with baseline characteristics revealed that women, younger participants, those with higher education, and physically active individuals showed a more positive attitude toward CAM. In contrast, male subjects were more open to genetic testing for the purpose of personalised therapy. Higher education levels as well as younger age were associated with less negative perceptions of conventional medication. The findings of this study suggest that attitudes towards medications and therapies may be influenced by gender, age and educational background. Further research is needed to explore how these attitudes towards conventional medication and alternative remedies might affect participation in cancer prevention.

## Supplementary Information

Additional file 1. Questionnaire on medications and therapies and their prescription.

Additional file 2. Baseline characteristics of the participants enrolled in the MIRACLE trial [15].

Additional file 3. Responses to the 25 questions, categorised according to the 5-point Likert scale and missing responses.

Additional file 4. Results of the exploratory analysis of factors with regard to the participant characteristics examined.

## Data Availability

The datasets used and/or analysed during the current study are available from the corresponding author on reasonable request.

## Abbreviations

ASA: Acetylsalicylic acid
CAM: Complementary and alternative medicine
ECOG: Eastern cooperative oncology group performance status
FCS: Fully conditional specification
GDP: Gross domestic product
