# Supplementary material for: Interest in complementary and alternative medicine among participants in a study on cancer prevention by green tea extract – results from an expert-based survey of MIRACLE trial participants
Source: BMC Complement Med Ther. 2025 Oct 2;25:350. doi: 10.1186/s12906-025-05087-3 (PMC12490030; doi:10.1186/s12906-025-05087-3)
Supplement: Supplementary file 1 — Additional file 1. Questionnaire on medications and therapies and their prescription. [file 12906_2025_5087_MOESM1_ESM.docx]

**Additional file 1: Questionnaire on medications and therapies and their prescription**

| **Question number** | **Wording** |
| --- | --- |
| **1** | Doctors prescribe too many medications. |
| **2** | People who take medications should take breaks from them occasionally. |
| **3** | Medications help many people live better lives. |
| **4** | Most medications are addictive. |
| **5** | Natural remedies are safer than medications. |
| **6** | In most cases, the benefits of medications outweigh the risks. |
| **7** | In the future, medications will be developed that can cure most diseases. |
| **8** | Medications do more harm than good. |
| **9** | Almost all medications are poison. |
| **10** | Medications extend the lives of many people. |
| **11** | Doctors trust medications too much. |
| **12** | If doctors had more time for their patients, they would prescribe fewer medications. |
| **13** | My body reacts very sensitively to medications. |
| **14** | I usually have a stronger reaction to medications than most people. |
| **15** | I have had bad reactions to medications in the past. |
| **16** | Even very small amounts of medication can overwhelm my body. |
| **17** | I would have my genes tested if it could tell me which medication is best for me. |
| **18** | Herbal medicines are more tolerable than chemically produced ones. |
| **19** | Herbal medicines are more effective than chemically produced ones. |
| **20** | If possible, I would prefer a herbal medication. |
| **21** | I am interested in alternative healing methods (e.g., homeopathy, acupuncture, traditional Chinese medicine, Ayurveda). |
| **22** | I regularly use alternative healing methods (e.g., acupuncture, homeopathic medicines, special therapies). |
| **23** | I see alternative healing methods as a meaningful complement to "classical conventional medicine." |
| **24** | I regularly take food or dietary supplements for prevention (e.g., probiotic foods). |
| **25** | Alternative healing methods are worth the financial investment. |

The questionnaire variables were presented on a 5-point Likert scale (1 = strongly disagree, 2 = disagree, 3 = neutral, 4 = agree, 5 = strongly agree).
