# Supplementary material for: Interest in complementary and alternative medicine among participants in a study on cancer prevention by green tea extract – results from an expert-based survey of MIRACLE trial participants
Source: BMC Complement Med Ther. 2025 Oct 2;25:350. doi: 10.1186/s12906-025-05087-3 (PMC12490030; doi:10.1186/s12906-025-05087-3)
Supplement: Supplementary file 2 — Additional file 2. Baseline characteristics of the participants enrolled in the MIRACLE trial [15]. [file 12906_2025_5087_MOESM2_ESM.docx]

**Additional file 2: Baseline characteristics of the participants enrolled in the MIRACLE trial [15]**

| **Characteristic** | | **Randomized participants with informed consent**  **(ITT-population)** | |
| --- | --- | --- | --- |
|  |  | **Green tea extract**  (n=429)^*^ | **Placebo**  (n=446)^*^ |
| **Sex,** no. (%)^**^ | Female  Male | 156 (36.4)  273 (63.6) | 169 (37.9)  277 (62.1) |
| **Age (years): median (range)** | | 64 (49-80) | 65 (45-80) |
| **Aspirin (≤100 mg/d),** no. (%)^**^ | Yes  No | 73 (17.0)  356 (83.0) | 71 (15.9)  375 (84.1) |
| **BMI (kg/m²): median (range)** | | 27.1 (17.5-43.4) | 27.1 (17.9-48.1) |
| **Excess bodyweight,** no. (%)^**^ | Overweight (BMI 25-29.9)  Obese (BMI≥30) | 175 (40.8)  114 (26.6) | 210 (47.1)  104 (23.3) |
| **Positive family history for CRC,**  no. (%)^**^ | Yes  No  n.a. | 108 (25.2)  313 (73.0)  8 (1.9) | 102 (22.9)  334 (74.9)  10 (2.2) |
| **Regular consumption of green tea,**  no. (%)^**^ | Yes  No  n.a. | 36 (8.4)  390 (90.9)  3 (0.7) | 38 (8.5)  405 (90.8)  3 (0.7) |
| **Nicotine consumption,** no. (%)^**^ | Present  Past  Never  n.a | 69 (16.1)  183 (42.7)  171 (39.9)  6 (1.4) | 75 (16.8)  158 (35.4)  204 (45.7)  9 (2.0) |
| **Alcohol consumption,** no. (%)^**^ | Yes  No  n.a. | 176 (41.0)  250 (58.3)  3 (0.7) | 191 (42.8)  252 (56.5)  3 (0.7) |
| **Regular exercise,** no. (%)^**^ | ≥ 30 min/week  ≥ 150 min/week  No  n.a. | 316 (73.7)  188 (43.8)  109 (25.4)  4 (0.9) | 344 (77.1)  207 (46.4)  98 (22.0)  4 (0.9) |
| **Colorectal adenoma characteristics** | Total number of adenomas ^***^  Number of adenomas per participant, mean (SD)^***^  Number of adenomas > 1cm per participant, mean (SD)^***^  Participants with ≥1 right-sided adenoma, no. (%)^*****^ | 876  2.08 (1.73)  0.36 (0.61)  274 (63.9) | 932  2.13 (2.03)  0.37 (0.62)  299 (67.0) |
| **Adenomas, histology**^****^, no. (%)^**^ | High grade IEN  Tubular  Villous  Tubulovillous  Serrated (SSA)  Advanced ^******^ | 26 (6.1)  326 (76.0)  12 (2.8)  82 (19.1)  69 (16.1)  162 (37.8) | 14 (3.1)  327 (73.3)  1 (0.2)  97 (21.7)  67 (15.0)  184 (41.3) |
| Abbreviations: BMI: body mass index; n.a. not assessed, IEN: intraepithelial neoplasia Body weight, body height, tobacco and alcolhol consumption, physical activity, family history for CRC were self-reported on a participant questionnaire. Data were missing for BMI for 10 randomized participants, for the number of adenomas for 2 and for the number of adenomas > 1cm for 70 randomized participants  * From three participants in the green tea group and one participant in the placebo group informed consent was not received and so their data are not reported here  ** Percentages do not always add up to 100 due to rounding  *** 8 participant in the green tea group and 7 participants in the placebo group without initial colorectal adenoma where excluded for the mean calculation as well as one placebo participant showing 27 adenomas  **** Categories are not mutually exclusive, several participants were qualified under more than one criterion.  ***** Right-sided adenoma is defined as adenoma located proximal to splenic flexure  ****** Advanced adenoma means at least one adenoma >1 cm and/or villous and/or tubulovillous and/or high-grade dysplasia and/or invasive | | | |
