# Supplementary material for: Interest in complementary and alternative medicine among participants in a study on cancer prevention by green tea extract – results from an expert-based survey of MIRACLE trial participants
Source: BMC Complement Med Ther. 2025 Oct 2;25:350. doi: 10.1186/s12906-025-05087-3 (PMC12490030; doi:10.1186/s12906-025-05087-3)
Supplement: Supplementary file 3 — Additional file 3. Responses to the 25 questions, categorised according to the 5-point Likert scale and missing responses. [file 12906_2025_5087_MOESM3_ESM.docx]

**Additional file 3: Responses to the 25 questions, categorised according to the 5-point Likert scale and missing responses**

| **Question number** | **n. a.** | | **Likert scale*** | | | | | | | | | | |
| --- | --- | --- | --- | --- | --- | --- | --- | --- | --- | --- | --- | --- | --- |
|  |  |  | **1** | | **2** | | **3** | | **4** | | **5** | |  |
|  | **n** | **%** | **n** | **%** | **n** | **%** | **n** | **%** | **n** | **%** | **n** | **%** | **Total (N)** |
| **1** | 12 | 1.3 | 69 | 7.4 | 318 | 34.0 | 319 | 34.1 | 183 | 20.0 | 34 | 3.6 | 935 |
| **2** | 15 | 1.6 | 106 | 11.3 | 282 | 30.2 | 270 | 28.9 | 227 | 24.3 | 35 | 2.7 | 935 |
| **3** | 10 | 1.1 | 16 | 1.7 | 35 | 3.7 | 53 | 5.7 | 580 | 62.0 | 241 | 25.8 | 935 |
| **4** | 6 | 0.6 | 101 | 10.8 | 389 | 41.6 | 334 | 35.7 | 90 | 9.6 | 15 | 1.6 | 935 |
| **5** | 6 | 0.6 | 49 | 5.2 | 301 | 32.2 | 392 | 41.9 | 153 | 16.4 | 34 | 3.6 | 935 |
| **6** | 10 | 1.1 | 11 | 1.2 | 71 | 7.6 | 180 | 19.3 | 586 | 62.7 | 77 | 8.2 | 935 |
| **7** | 14 | 1.5 | 8 | 0.9 | 131 | 14.0 | 324 | 34.7 | 396 | 42.4 | 62 | 6.6 | 935 |
| **8** | 33 | 3.5 | 113 | 12.1 | 586 | 62.7 | 165 | 17.6 | 32 | 3.4 | 6 | 0.6 | 935 |
| **9** | 9 | 1.0 | 127 | 13.6 | 479 | 51.2 | 194 | 20.7 | 114 | 12.2 | 12 | 1.3 | 935 |
| **10** | 9 | 1.0 | 12 | 1.3 | 45 | 4.8 | 49 | 5.2 | 620 | 66.3 | 200 | 21.4 | 935 |
| **11** | 7 | 0.7 | 18 | 1.9 | 200 | 21.4 | 426 | 45.6 | 246 | 26.3 | 38 | 4.1 | 935 |
| **12** | 4 | 0.4 | 25 | 2.7 | 172 | 18.4 | 232 | 24.8 | 381 | 40.7 | 121 | 12.9 | 935 |
| **13** | 11 | 1.2 | 76 | 8.1 | 416 | 44.5 | 203 | 21.7 | 176 | 18.8 | 53 | 5.7 | 935 |
| **14** | 9 | 1.0 | 116 | 12.4 | 415 | 44.4 | 317 | 33.9 | 53 | 5.7 | 25 | 2.7 | 935 |
| **15** | 9 | 1.0 | 149 | 15.3 | 481 | 51.4 | 105 | 11.2 | 174 | 18.6 | 17 | 1.8 | 935 |
| **16** | 7 | 0.7 | 204 | 21.8 | 519 | 55.5 | 143 | 15.3 | 48 | 5.1 | 14 | 1.5 | 935 |
| **17** | 10 | 1.1 | 23 | 2.5 | 114 | 12.2 | 161 | 17.2 | 444 | 47.5 | 183 | 19.6 | 935 |
| **18** | 10 | 1.1 | 16 | 1.7 | 137 | 14.7 | 339 | 36.3 | 364 | 38.9 | 69 | 7.4 | 935 |
| **19** | 13 | 1.4 | 23 | 2.5 | 241 | 25.8 | 552 | 59.0 | 91 | 9.7 | 15 | 1.6 | 935 |
| **20** | 14 | 1.5 | 10 | 1.1 | 100 | 10.7 | 143 | 15.3 | 542 | 58.0 | 126 | 13.5 | 935 |
| **21** | 18 | 1.9 | 41 | 4.4 | 216 | 23.1 | 111 | 11.9 | 432 | 46.2 | 117 | 12.5 | 935 |
| **22** | 23 | 2.5 | 188 | 20.1 | 508 | 54.3 | 56 | 6.0 | 130 | 13.9 | 30 | 3.2 | 935 |
| **23** | 12 | 1.3 | 11 | 1.2 | 60 | 6.4 | 140 | 15.0 | 507 | 54.2 | 205 | 21.9 | 935 |
| **24** | 14 | 1.5 | 198 | 21.2 | 510 | 54.5 | 44 | 4.7 | 142 | 15.2 | 27 | 2.9 | 935 |
| **25** | 11 | 1.2 | 32 | 3.4 | 109 | 11.7 | 425 | 45.5 | 291 | 31.1 | 67 | 7.2 | 935 |
| **Total (N)** | 296 |  | 1742 |  | 6835 |  | 5677 |  | 7002 |  | 1823 |  |  |

* The 5 points of the Likert scale are: 1 = strongly disagree, 2 = disagree, 3 = neutral, 4 = agree, 5 = strongly agree. Abbreviations: n. a., not available (missing response)
