# Supplementary material for: Interest in complementary and alternative medicine among participants in a study on cancer prevention by green tea extract – results from an expert-based survey of MIRACLE trial participants
Source: BMC Complement Med Ther. 2025 Oct 2;25:350. doi: 10.1186/s12906-025-05087-3 (PMC12490030; doi:10.1186/s12906-025-05087-3)
Supplement: Supplementary file 4 — Additional file 4. Results of the exploratory analysis of factors with regard to the participant characteristics examined. [file 12906_2025_5087_MOESM4_ESM.docx]

**Additional file 4: Results of the exploratory analysis of factors with regard to the participant characteristics examined**

| **Participant characteristic** | **Category** | **Factor*** | **N** | **N**  **Miss** | **Min** | **Max** | **Lower Quartile** | **Upper Quartile** | **Median** | **Mean** | **SD** |
| --- | --- | --- | --- | --- | --- | --- | --- | --- | --- | --- | --- |
| Gender | male | 1 | 574 | 4 | 1.00 | 4.50 | 2.00 | 2.75 | 2.25 | 2.30 | 0.66 |
|  |  | 2 | 574 | 4 | 1.00 | 5.00 | 3.00 | 4.00 | 3.67 | 3.45 | 0.71 |
|  |  | 3 | 575 | 3 | 1.00 | 5.00 | 3.00 | 3.50 | 3.25 | 3.20 | 0.61 |
|  |  | 4 | 577 | 1 | 1.00 | 5.00 | 3.50 | 4.00 | 3.75 | 3.81 | 0.52 |
|  |  | 5 | 578 | 0 | 1.00 | 5.00 | 2.67 | 3.67 | 3.00 | 3.09 | 0.72 |
|  |  | 6 | 577 | 1 | 1.00 | 4.75 | 2.00 | 2.75 | 2.50 | 2.47 | 0.61 |
|  |  | 7 | 571 | 7 | 1.00 | 5.00 | 2.00 | 2.50 | 2.00 | 2.16 | 0.78 |
|  |  | 8 | 572 | 6 | 1.00 | 5.00 | 3.00 | 4.00 | 4.00 | 3.76 | 0.96 |
|  | female | 1 | 355 | 2 | 1.00 | 5.00 | 2.00 | 3.00 | 2.50 | 2.55 | 0.83 |
|  |  | 2 | 353 | 4 | 1.00 | 5.00 | 3.00 | 4.00 | 3.67 | 3.64 | 0.82 |
|  |  | 3 | 355 | 1 | 1.00 | 5.00 | 2.75 | 3.50 | 3.25 | 3.15 | 0.68 |
|  |  | 4 | 356 | 1 | 1.75 | 5.00 | 3.50 | 4.00 | 3.75 | 3.78 | 0.51 |
|  |  | 5 | 355 | 2 | 1.00 | 5.00 | 2.67 | 3.67 | 3.00 | 3.12 | 0.70 |
|  |  | 6 | 355 | 2 | 1.00 | 5.24 | 2.00 | 2.75 | 2.50 | 2.42 | 0.63 |
|  |  | 7 | 354 | 3 | 1.00 | 5.00 | 2.00 | 3.00 | 2.00 | 2.35 | 0.96 |
|  |  | 8 | 353 | 4 | 1.00 | 5.00 | 3.00 | 4.00 | 4.00 | 3.61 | 1.06 |
| Age | < 55 years | 1 | 115 | 1 | 1.00 | 4.75 | 2.00 | 3.00 | 2.25 | 2.41 | 0.78 |
|  |  | 2 | 113 | 3 | 1.67 | 5.00 | 3.33 | 4.00 | 4.00 | 3.79 | 0.68 |
|  |  | 3 | 113 | 3 | 1.75 | 5.00 | 2.75 | 3.50 | 3.00 | 3.16 | 0.63 |
|  |  | 4 | 115 | 1 | 2.00 | 5.00 | 3.50 | 4.00 | 3.75 | 3.77 | 0.53 |
|  |  | 5 | 115 | 1 | 1.00 | 5.00 | 2.67 | 3.50 | 3.00 | 3.05 | 0.73 |
|  |  | 6 | 115 | 1 | 1.00 | 3.75 | 2.00 | 2.75 | 2.25 | 2.29 | 0.62 |
|  |  | 7 | 113 | 3 | 1.00 | 4.50 | 1.75 | 3.00 | 2.00 | 2.27 | 0.85 |
|  |  | 8 | 113 | 3 | 1.00 | 5.00 | 3.00 | 4.00 | 4.00 | 3.75 | 1.00 |
|  | ≥ 55 - 74 years | 1 | 730 | 4 | 1.00 | 5.00 | 2.00 | 2.75 | 2.25 | 2.38 | 0.75 |
|  |  | 2 | 731 | 3 | 1.00 | 5.00 | 3.00 | 4.00 | 3.67 | 3.48 | 0.78 |
|  |  | 3 | 732 | 2 | 1.00 | 5.00 | 2.75 | 3.50 | 3.25 | 3.18 | 0.63 |
|  |  | 4 | 733 | 1 | 1.00 | 5.00 | 3.50 | 4.00 | 3.75 | 3.81 | 0.51 |
|  |  | 5 | 733 | 1 | 1.00 | 5.00 | 2.67 | 3.67 | 3.00 | 3.11 | 0.72 |
|  |  | 6 | 732 | 2 | 1.00 | 4.75 | 2.00 | 3.00 | 2.50 | 2.47 | 0.62 |
|  |  | 7 | 728 | 6 | 1.00 | 5.00 | 1.63 | 3.00 | 2.00 | 2.21 | 0.85 |
|  |  | 8 | 730 | 4 | 1.00 | 5.00 | 3.00 | 4.00 | 4.00 | 3.71 | 0.99 |
|  | ≥ 75 years | 1 | 84 | 1 | 1.00 | 4.25 | 2.00 | 2.75 | 2.50 | 2.47 | 0.67 |
|  |  | 2 | 83 | 2 | 2.00 | 5.00 | 3.33 | 4.00 | 3.67 | 3.57 | 0.66 |
|  |  | 3 | 85 | 0 | 1.00 | 4.75 | 3.00 | 3.50 | 3.25 | 3.18 | 0.67 |
|  |  | 4 | 85 | 0 | 1.50 | 5.00 | 3.50 | 4.00 | 3.75 | 3.78 | 0.53 |
|  |  | 5 | 85 | 0 | 1.00 | 5.00 | 2.67 | 3.33 | 3.00 | 3.06 | 0.64 |
|  |  | 6 | 85 | 0 | 1.00 | 4.00 | 2.00 | 2.75 | 2.50 | 2.47 | 0.54 |
|  |  | 7 | 84 | 1 | 1.00 | 5.00 | 2.00 | 3.00 | 2.00 | 2.34 | 0.89 |
|  |  | 8 | 82 | 3 | 1.00 | 5.00 | 3.00 | 4.00 | 4.00 | 3.60 | 1.12 |
| low-dose ASA | no | 1 | 782 | 5 | 1.00 | 5.00 | 2.00 | 2.75 | 2.25 | 2.39 | 0.75 |
|  |  | 2 | 780 | 7 | 1.00 | 5.00 | 3.00 | 4.00 | 3.67 | 3.55 | 0.76 |
|  |  | 3 | 782 | 5 | 1.00 | 5.00 | 2.75 | 3.50 | 3.25 | 3.19 | 0.65 |
|  |  | 4 | 785 | 2 | 1.00 | 5.00 | 3.50 | 4.00 | 3.75 | 3.79 | 0.52 |
|  |  | 5 | 785 | 2 | 1.00 | 5.00 | 2.67 | 3.67 | 3.00 | 3.10 | 0.71 |
|  |  | 6 | 784 | 3 | 1.00 | 4.75 | 2.00 | 2.75 | 2.50 | 2.46 | 0.62 |
|  |  | 7 | 777 | 10 | 1.00 | 5.00 | 1.50 | 3.00 | 2.00 | 2.22 | 0.86 |
|  |  | 8 | 777 | 10 | 1.00 | 5.00 | 3.00 | 4.00 | 4.00 | 3.69 | 1.01 |
|  | yes | 1 | 147 | 1 | 1.00 | 4.25 | 2.00 | 2.75 | 2.25 | 2.40 | 0.70 |
|  |  | 2 | 147 | 1 | 1.00 | 5.00 | 3.00 | 4.00 | 3.33 | 3.41 | 0.76 |
|  |  | 3 | 148 | 0 | 1.75 | 5.00 | 3.75 | 3.50 | 3.25 | 3.13 | 0.59 |
|  |  | 4 | 148 | 0 | 2.00 | 5.00 | 3.54 | 4.25 | 4.00 | 3.87 | 0.49 |
|  |  | 5 | 148 | 0 | 1.00 | 5.00 | 2.67 | 3.67 | 3.00 | 3.09 | 0.72 |
|  |  | 6 | 148 | 0 | 1.00 | 4.00 | 2.00 | 2.75 | 2.33 | 2.40 | 0.59 |
|  |  | 7 | 148 | 0 | 1.00 | 5.00 | 2.00 | 3.00 | 2.00 | 2.30 | 0.81 |
|  |  | 8 | 148 | 0 | 1.00 | 5.00 | 3.00 | 4.00 | 4.00 | 3.78 | 0.97 |
| Adenoma >1cm | No adenoma >1cm | 1 | 585 | 3 | 1.00 | 5.00 | 2.00 | 2.75 | 2.25 | 2.40 | 0.74 |
|  |  | 2 | 582 | 6 | 1.00 | 5.00 | 3.00 | 4.00 | 3.67 | 3.52 | 0.76 |
|  |  | 3 | 585 | 3 | 1.00 | 5.00 | 2.75 | 3.50 | 3.25 | 3.19 | 0.63 |
|  |  | 4 | 586 | 2 | 1.00 | 5.00 | 3.50 | 4.00 | 3.75 | 3.81 | 0.50 |
|  |  | 5 | 586 | 2 | 1.00 | 5.00 | 2.67 | 3.67 | 3.00 | 3.13 | 0.72 |
|  |  | 6 | 585 | 3 | 1.00 | 4.25 | 2.00 | 2.75 | 2.50 | 2.45 | 0.61 |
|  |  | 7 | 581 | 7 | 1.00 | 5.00 | 2.00 | 3.00 | 2.00 | 2.23 | 0.86 |
|  |  | 8 | 581 | 1 | 1.00 | 5.00 | 3.00 | 4.00 | 4.00 | 3.71 | 1.00 |
|  | ≥1 adenoma >1cm | 1 | 264 | 1 | 1.00 | 5.00 | 2.00 | 3.00 | 2.25 | 2.40 | 0.75 |
|  |  | 2 | 263 | 2 | 1.00 | 5.00 | 3.00 | 4.00 | 3.67 | 3.58 | 0.75 |
|  |  | 3 | 263 | 2 | 1.00 | 5.00 | 3.00 | 3.50 | 3.25 | 3.20 | 0.66 |
|  |  | 4 | 265 | 0 | 1.75 | 5.00 | 3.50 | 4.00 | 3.75 | 3.79 | 0.51 |
|  |  | 5 | 265 | 0 | 1.00 | 5.00 | 2.67 | 3.67 | 3.00 | 3.09 | 0.72 |
|  |  | 6 | 265 | 0 | 1.00 | 4.00 | 2.00 | 3.00 | 2.50 | 2.46 | 0.63 |
|  |  | 7 | 263 | 2 | 1.00 | 5.00 | 1.50 | 3.00 | 2.00 | 2.22 | 0.85 |
|  |  | 8 | 262 | 3 | 1.00 | 5.00 | 3.00 | 4.00 | 4.00 | 3.73 | 1.02 |
|  | n.a. | 1 | 80 | 2 | 1.00 | 4.75 | 2.00 | 2.75 | 2.13 | 2.34 | 0.76 |
|  |  | 2 | 82 | 0 | 1.00 | 5.00 | 3.00 | 4.00 | 3.33 | 3.36 | 0.77 |
|  |  | 3 | 82 | 0 | 1.00 | 4.50 | 2.75 | 3.50 | 3.00 | 3.03 | 0.59 |
|  |  | 4 | 82 | 0 | 1.75 | 5.00 | 3.50 | 4.00 | 4.00 | 3.76 | 0.58 |
|  |  | 5 | 82 | 0 | 1.33 | 4.67 | 2.58 | 3.33 | 3.00 | 2.96 | 0.65 |
|  |  | 6 | 82 | 0 | 1.00 | 4.75 | 2.00 | 2.75 | 2.29 | 2.37 | 0.63 |
|  |  | 7 | 81 | 0 | 1.00 | 5.00 | 2.00 | 3.00 | 2.00 | 2.30 | 0.86 |
|  |  | 8 | 82 | 0 | 1.00 | 5.00 | 3.00 | 4.00 | 4.00 | 3.57 | 0.97 |
| Region | East G | 1 | 210 | 1 | 1.00 | 4.75 | 2.00 | 2.75 | 2.25 | 2.37 | 0.76 |
|  |  | 2 | 210 | 1 | 1.00 | 5.00 | 3.00 | 4.00 | 3.33 | 3.41 | 0.73 |
|  |  | 3 | 210 | 1 | 1.00 | 5.00 | 3.00 | 3.50 | 3.25 | 3.24 | 0.67 |
|  |  | 4 | 211 | 0 | 1.50 | 5.00 | 3.50 | 4.00 | 3.75 | 3.74 | 0.57 |
|  |  | 5 | 211 | 0 | 1.00 | 5.00 | 2.33 | 3.67 | 3.00 | 3.00 | 0.79 |
|  |  | 6 | 211 | 0 | 1.00 | 4.25 | 2.00 | 2.75 | 2.50 | 2.43 | 0.62 |
|  |  | 7 | 209 | 2 | 1.00 | 4.50 | 2.00 | 2.50 | 2.00 | 2.14 | 0.74 |
|  |  | 8 | 209 | 2 | 1.00 | 5.00 | 3.00 | 4.00 | 4.00 | 3.62 | 0.98 |
|  | West G | 1 | 328 | 2 | 1.00 | 5.00 | 2.00 | 2.75 | 2.25 | 2.38 | 0.78 |
|  |  | 2 | 326 | 4 | 1.00 | 5.00 | 3.00 | 4.00 | 3.67 | 3.60 | 0.77 |
|  |  | 3 | 328 | 2 | 1.00 | 5.00 | 2.75 | 3.50 | 3.25 | 3.16 | 0.66 |
|  |  | 4 | 330 | 0 | 2.00 | 5.00 | 3.50 | 4.00 | 4.00 | 3.83 | 0.48 |
|  |  | 5 | 330 | 0 | 1.00 | 5.00 | 2.67 | 3.67 | 3.00 | 3.11 | 0.67 |
|  |  | 6 | 330 | 0 | 1.00 | 4.00 | 2.00 | 2.75 | 2.50 | 2.42 | 0.61 |
|  |  | 7 | 327 | 3 | 1.00 | 5.00 | 2.00 | 3.00 | 2.00 | 2.27 | 0.89 |
|  |  | 8 | 327 | 3 | 1.00 | 5.00 | 3.00 | 4.00 | 4.00 | 3.74 | 1.00 |
|  | Southern G | 1 | 391 | 3 | 1.00 | 5.00 | 2.00 | 3.00 | 2.25 | 2.42 | 0.70 |
|  |  | 2 | 391 | 3 | 1.00 | 5.00 | 3.00 | 4.00 | 3.67 | 3.52 | 0.76 |
|  |  | 3 | 392 | 2 | 1.00 | 5.00 | 2.75 | 3.50 | 3.25 | 3.16 | 0.60 |
|  |  | 4 | 392 | 2 | 1.00 | 5.00 | 3.50 | 4.00 | 3.88 | 3.81 | 0.51 |
|  |  | 5 | 392 | 2 | 1.00 | 5.00 | 2.67 | 3.67 | 3.00 | 3.14 | 0.70 |
|  |  | 6 | 391 | 3 | 1.00 | 4.75 | 2.00 | 3.00 | 2.50 | 2.49 | 0.61 |
|  |  | 7 | 389 | 5 | 1.00 | 5.00 | 1.50 | 3.00 | 2.00 | 2.25 | 0.89 |
|  |  | 8 | 389 | 5 | 1.00 | 5.00 | 3.00 | 4.00 | 4.00 | 3.72 | 1.01 |
| Regular exercise | No regular exercise | 1 | 214 | 2 | 1.00 | 5.00 | 2.00 | 3.00 | 2.25 | 2.40 | 0.75 |
|  |  | 2 | 216 | 0 | 1.00 | 5.00 | 2.67 | 4.00 | 3.33 | 3.36 | 0.76 |
|  |  | 3 | 216 | 0 | 1.00 | 5.00 | 3.00 | 3.50 | 3.25 | 3.17 | 0.65 |
|  |  | 4 | 216 | 0 | 1.75 | 5.00 | 3.50 | 4.00 | 3.75 | 3.78 | 0.57 |
|  |  | 5 | 216 | 0 | 1.00 | 5.00 | 2.67 | 3.33 | 3.00 | 2.97 | 0.77 |
|  |  | 6 | 216 | 0 | 1.00 | 4.75 | 2.00 | 2.75 | 2.50 | 2.45 | 0.64 |
|  |  | 7 | 213 | 3 | 1.00 | 5.00 | 1.50 | 2.50 | 2.00 | 2.11 | 0.75 |
|  |  | 8 | 214 | 2 | 1.00 | 5.00 | 3.00 | 4.00 | 4.00 | 3.57 | 1.07 |
|  | Regular exercise ≤3 hours per week | 1 | 379 | 3 | 1.00 | 5.00 | 2.00 | 3.00 | 2.50 | 2.43 | 0.71 |
|  |  | 2 | 376 | 6 | 1.00 | 5.00 | 3.00 | 4.00 | 3.67 | 3.60 | 0.75 |
|  |  | 3 | 378 | 4 | 1.25 | 5.00 | 2.75 | 3.50 | 3.25 | 3.15 | 0.60 |
|  |  | 4 | 380 | 2 | 2.00 | 5.00 | 3.50 | 4.00 | 3.75 | 3.84 | 0.46 |
|  |  | 5 | 380 | 2 | 1.00 | 5.00 | 2.67 | 3.67 | 3.00 | 3.15 | 0.68 |
|  |  | 6 | 379 | 3 | 1.00 | 4.25 | 2.00 | 2.75 | 2.50 | 2.45 | 0.60 |
|  |  | 7 | 377 | 5 | 1.00 | 5.00 | 2.00 | 3.00 | 2.00 | 2.32 | 0.89 |
|  |  | 8 | 377 | 5 | 1.00 | 5.00 | 3.00 | 4.00 | 4.00 | 3.70 | 1.00 |
|  | Regular exercise >3 hours per week | 1 | 321 | 1 | 1.00 | 4.75 | 2.00 | 2.75 | 2.25 | 2.34 | 0.78 |
|  |  | 2 | 320 | 2 | 1.33 | 5.00 | 3.00 | 4.00 | 3.67 | 3.56 | 0.77 |
|  |  | 3 | 321 | 1 | 1.00 | 5.00 | 2.75 | 3.50 | 3.25 | 3.21 | 0.68 |
|  |  | 4 | 322 | 0 | 1.00 | 5.00 | 3.50 | 4.00 | 3.75 | 3.78 | 0.52 |
|  |  | 5 | 322 | 0 | 1.67 | 5.00 | 2.67 | 3.67 | 3.00 | 3.14 | 0.69 |
|  |  | 6 | 322 | 0 | 1.00 | 4.25 | 2.00 | 2.75 | 2.50 | 2.46 | 0.62 |
|  |  | 7 | 320 | 2 | 1.00 | 5.00 | 1.50 | 3.00 | 2.00 | 2.21 | 0.87 |
|  |  | 8 | 319 | 3 | 1.00 | 5.00 | 3.00 | 4.00 | 4.00 | 3.77 | 0.95 |
|  | n.a. | 1 | 15 | 0 | 1.00 | 3.33 | 2.00 | 3.00 | 2.75 | 2.51 | 0.68 |
|  |  | 2 | 15 | 0 | 1.67 | 4.00 | 3.00 | 4.00 | 3.33 | 3.36 | 0.68 |
|  |  | 3 | 15 | 0 | 2.33 | 4.50 | 3.00 | 3.50 | 3.25 | 3.32 | 0.55 |
|  |  | 4 | 15 | 0 | 1.50 | 5.00 | 3.50 | 4.00 | 3.75 | 3.68 | 0.78 |
|  |  | 5 | 15 | 0 | 1.67 | 4.33 | 2.33 | 4.00 | 3.00 | 2.93 | 0.84 |
|  |  | 6 | 15 | 0 | 1.00 | 3.25 | 2.00 | 3.25 | 2.25 | 2.32 | 0.69 |
|  |  | 7 | 15 | 0 | 1.00 | 4.50 | 2.00 | 2.50 | 2.00 | 2.30 | 0.94 |
|  |  | 8 | 15 | 0 | 2.00 | 5.00 | 4.00 | 5.00 | 4.00 | 4.27 | 0.80 |
| Educational qualification | Lower sec. school | 1 | 351 | 2 | 1.00 | 4.75 | 2.00 | 3.00 | 2.25 | 2.46 | 0.73 |
|  |  | 2 | 351 | 2 | 1.00 | 5.00 | 3.00 | 4.00 | 3.33 | 3.43 | 0.76 |
|  |  | 3 | 353 | 0 | 1.75 | 5.00 | 3.00 | 3.50 | 3.25 | 3.28 | 0.56 |
|  |  | 4 | 353 | 0 | 1.75 | 5.00 | 3.50 | 4.00 | 4.00 | 3.81 | 0.52 |
|  |  | 5 | 353 | 0 | 1.00 | 5.00 | 2.67 | 3.67 | 3.00 | 3.06 | 0.68 |
|  |  | 6 | 353 | 0 | 1.00 | 4.75 | 2.25 | 3.00 | 2.50 | 2.55 | 0.61 |
|  |  | 7 | 351 | 2 | 1.00 | 5.00 | 2.00 | 3.00 | 2.00 | 2.23 | 0.83 |
|  |  | 8 | 353 | 0 | 1.00 | 5.00 | 3.00 | 4.00 | 4.00 | 3.73 | 0.98 |
|  | Intermed. sec. school | 1 | 271 | 1 | 1.00 | 5.00 | 2.00 | 3.00 | 2.50 | 2.43 | 0.77 |
|  |  | 2 | 268 | 4 | 1.00 | 5.00 | 3.00 | 4.00 | 3.67 | 3.52 | 0.76 |
|  |  | 3 | 269 | 3 | 1.00 | 5.00 | 2.75 | 3.50 | 3.25 | 3.14 | 0.67 |
|  |  | 4 | 272 | 0 | 1.75 | 5.00 | 2.50 | 4.00 | 3.75 | 3.78 | 0.48 |
|  |  | 5 | 272 | 0 | 1.00 | 6.00 | 2.67 | 3.67 | 3.00 | 3.12 | 0.73 |
|  |  | 6 | 272 | 0 | 1.00 | 4.25 | 2.00 | 3.00 | 2.50 | 2.48 | 0.60 |
|  |  | 7 | 266 | 6 | 1.00 | 5.00 | 2.00 | 3.00 | 2.00 | 2.23 | 0.86 |
|  |  | 8 | 266 | 6 | 1.00 | 5.00 | 3.00 | 4.00 | 4.00 | 3.62 | 1.03 |
|  | High school | 1 | 59 | 2 | 1.00 | 4.00 | 2.00 | 2.50 | 2.25 | 2.28 | 0.70 |
|  |  | 2 | 59 | 2 | 1.67 | 5.00 | 3.00 | 4.00 | 3.67 | 3.55 | 0.68 |
|  |  | 3 | 59 | 2 | 1.75 | 4.50 | 2.50 | 3.50 | 3.00 | 3.05 | 0.70 |
|  |  | 4 | 60 | 1 | 2.67 | 5.00 | 3.50 | 4.00 | 3.75 | 3.86 | 0.50 |
|  |  | 5 | 59 | 2 | 1.67 | 5.00 | 2.33 | 3.67 | 3.00 | 3.03 | 0.79 |
|  |  | 6 | 59 | 2 | 1.00 | 4.25 | 2.00 | 2.75 | 2.50 | 2.38 | 0.65 |
|  |  | 7 | 60 | 1 | 1.00 | 5.00 | 1.50 | 2.50 | 2.00 | 2.08 | 0.86 |
|  |  | 8 | 59 | 2 | 1.00 | 5.00 | 3.00 | 4.00 | 4.00 | 3.56 | 0.97 |
|  | University degree | 1 | 238 | 1 | 1.00 | 4.75 | 1.94 | 2.75 | 2.25 | 2.30 | 0.72 |
|  |  | 2 | 239 | 0 | 1.33 | 5.00 | 3.33 | 4.00 | 3.67 | 3.67 | 0.75 |
|  |  | 3 | 239 | 0 | 1.00 | 5.00 | 2.75 | 3.50 | 3.25 | 3.11 | 0.67 |
|  |  | 4 | 238 | 1 | 1.00 | 5.00 | 3.50 | 4.00 | 3.75 | 3.81 | 0.53 |
|  |  | 5 | 239 | 0 | 1.00 | 5.00 | 2.67 | 3.67 | 3.00 | 3.17 | 0.71 |
|  |  | 6 | 238 | 1 | 1.00 | 4.00 | 2.00 | 2.67 | 2.25 | 2.29 | 0.60 |
|  |  | 7 | 238 | 1 | 1.00 | 5.00 | 1.50 | 3.00 | 2.00 | 2.26 | 0.89 |
|  |  | 8 | 237 | 2 | 1.00 | 5.00 | 3.00 | 4.00 | 4.00 | 3.78 | 0.99 |
|  | n.a. | 1 | 10 | 0 | 1.00 | 4.00 | 1.44 | 2.31 | 1.88 | 2.00 | 0.85 |
|  |  | 2 | 10 | 0 | 1.00 | 4.67 | 2.83 | 1.08 | 3.42 | 3.28 | 1.05 |
|  |  | 3 | 10 | 0 | 1.75 | 4.50 | 2.19 | 3.63 | 3.13 | 3.02 | 0.87 |
|  |  | 4 | 10 | 0 | 1.50 | 4.50 | 3.50 | 4.00 | 3.88 | 3.65 | 0.81 |
|  |  | 5 | 10 | 0 | 1.33 | 4.67 | 2.25 | 3.75 | 2.67 | 2.87 | 1.00 |
|  |  | 6 | 10 | 0 | 1.00 | 3.50 | 1.88 | 2.63 | 2.13 | 2.23 | 0.71 |
|  |  | 7 | 10 | 0 | 1.00 | 4.50 | 1.75 | 3.00 | 2.00 | 2.25 | 1.03 |
|  |  | 8 | 10 | 0 | 2.00 | 5.00 | 2.75 | 5.00 | 4.00 | 3.80 | 1.14 |
| Adherence | adherent | 1 | 611 | 4 | 1.00 | 5.00 | 2.00 | 2.75 | 2.25 | 2.34 | 0.71 |
|  |  | 2 | 610 | 5 | 1.00 | 5.00 | 3.00 | 4.00 | 3.67 | 3.52 | 0.77 |
|  |  | 3 | 612 | 3 | 1.00 | 5.00 | 2.75 | 3.50 | 3.25 | 3.19 | 0.63 |
|  |  | 4 | 614 | 1 | 1.00 | 5.00 | 3.50 | 4.00 | 3.75 | 3.80 | 0.52 |
|  |  | 5 | 614 | 1 | 1.00 | 5.00 | 2.67 | 3.67 | 3.00 | 3.07 | 0.69 |
|  |  | 6 | 614 | 1 | 1.00 | 4.75 | 2.00 | 2.75 | 2.50 | 2.43 | 0.61 |
|  |  | 7 | 608 | 7 | 1.00 | 5.00 | 1.50 | 3.00 | 2.00 | 2.21 | 0.86 |
|  |  | 8 | 608 | 7 | 1.00 | 5.00 | 3.00 | 4.00 | 4.00 | 3.69 | 0.99 |
|  | non-adherent | 1 | 214 | 2 | 1.00 | 5.00 | 2.00 | 3.00 | 2.25 | 2.41 | 0.77 |
|  |  | 2 | 213 | 3 | 1.67 | 5.00 | 3.00 | 4.00 | 3.67 | 3.52 | 0.76 |
|  |  | 3 | 214 | 2 | 1.25 | 5.00 | 2.75 | 2.50 | 3.00 | 3.14 | 0.64 |
|  |  | 4 | 215 | 1 | 1.75 | 5.00 | 3.50 | 4.00 | 3.75 | 3.82 | 0.49 |
|  |  | 5 | 215 | 1 | 1.00 | 5.00 | 2.67 | 3.67 | 3.00 | 3.12 | 0.77 |
|  |  | 6 | 214 | 2 | 1.00 | 4.25 | 2.00 | 3.00 | 2.50 | 2.47 | 0.63 |
|  |  | 7 | 213 | 3 | 1.00 | 5.00 | 2.00 | 2.75 | 2.00 | 2.22 | 0.78 |
|  |  | 8 | 213 | 3 | 1.00 | 5.00 | 3.00 | 4.00 | 4.00 | 3.77 | 1.01 |

* The mean values (Mean) refer to the score achieved on the 5-point Likert scale, which ranges from 1 = strongly disagree to 5 = strongly agree. The following additional values are given for the eight factors: Total number of participants considered for the factor analysis (N), total number of participants excluded from the factor analysis (N Miss), minimum (Min), maximum (Max), lower quartiles (Lower Quartile) and upper quartiles (Upper Quartile), median, and standard deviation (SD). Further abbreviations: ASA, acetylsalicylic acid; intermed., intermediate; n.a., missing information; sec., secondary.
